# Three-Dimensional Modeling of the Optical Switch Based on Guided-Mode Resonances in Photonic Crystals

**DOI:** 10.3390/mi14061116

**Published:** 2023-05-26

**Authors:** Atiq Ur Rehman, Yousuf Khan, Muhammad Irfan, Shahzaib Choudri, Svetlana N. Khonina, Nikolay L. Kazanskiy, Muhammad A. Butt

**Affiliations:** 1 Nanophotonics Research Group, Department of Electronic Engineering, Balochistan University of Information Technology, Engineering and Management Sciences, Quetta 87300, Pakistan; 2 Department of Technical Cybernetics, Samara National Research University, 443086 Samara, Russiabutt.m@ssau.ru (M.A.B.); 3 IPSI RAS-Branch of the FSRC “Crystallography and Photonics” RAS, 443001 Samara, Russia

**Keywords:** optical switch, guided mode resonances, finite difference time domain, photonic crystals, optical amplification

## Abstract

Optical switching is an essential part of photonic integrated circuits and the focus of research at the moment. In this research, an optical switch design working on the phenomenon of guided-mode resonances in a 3D photonic-crystal-based structure is reported. The optical-switching mechanism is studied in a dielectric slab-waveguide-based structure operating in the near-infrared range in a telecom window of 1.55 µm. The mechanism is investigated via the interference of two signals, i.e., the data signal and the control signal. The data signal is coupled into the optical structure and filtered utilizing guided-mode resonance, whereas the control signal is index-guided in the optical structure. The amplification or de-amplification of the data signal is controlled by tuning the spectral properties of the optical sources and structural parameters of the device. The parameters are optimized first using a single-cell model with periodic boundary conditions and later in a finite 3D-FDTD model of the device. The numerical design is computed in an open-source Finite Difference Time Domain simulation platform. Optical amplification in the range of 13.75% is achieved in the data signal with a decrease in the linewidth up to 0.0079 µm, achieving a quality factor of 114.58. The proposed device presents great potential in the field of photonic integrated circuits, biomedical technology, and programmable photonics.

## 1. Introduction

Photonic technology has undergone a boost in the last couple of decades and has come forward to replace electronic technology in various applications due to its power efficiency and ability to operate in Terahertz (THz) frequency ranges. Specifically, the optical integrated components such as switches [1], modulators [2], multiplexers [3], logic gates [4,5], and filters [6], which play an essential role in an optical integrated circuit, have become the center of research nowadays. Researchers are busy digging down the architecture and topology of photonic integrated circuits (PICs), keeping in view the process-related complexity, performance, and fabrication as a whole. Considering optical technology as the best alternative to electronic technology [7], the optical fields are brought together to use different phenomena such as interference [8], guided-mode resonances (GMR) [9], the Kerr effect [10], and many more [11]. Moreover, the proper choice of materials is a key factor in designing these optical devices. Therefore, among periodic nanostructures, photonic crystals (PhCs) have recently brought us a step closer to the achievement of optical integrated circuits. These PhCs are periodic dielectric nanostructures that are capable of controlling light at the wavelength scale [12]. The concept of the PhCs is derived from nature, presenting themselves on the bodies of different living organisms, i.e., insects, parrots, peacocks, and stones [13]. They exhibit different phenomena of light in terms of reflection, refraction, diffraction, and total internal reflection [14]. PhCs can be created artificially to design, produce, and achieve what is required of a certain component or a device in the real world [15]. 

Considering the components of a PIC, an optical switch is an important element that can work analogous to the electronic transistor in electronic integrated circuits. Therefore, utmost efforts are made to achieve the functions of optical switching elements using the time domain [16] or frequency domain [17], with different topologies in the form of cross waveguide geometries [18], quantum dots [19], optoelectronic hybrid devices [20], ring resonators [21], combined configurations of thermodynamics and optical components [22], and the utilization of transparent and active materials [23,24], with its desired function and implementation in optical circuits. Therefore, a 2D-FDTD design of an optical switch is investigated in [25], utilizing the phenomenon of GMR with varying radius cavities implemented at the start of the PhC-mesh. Another approach uses GMR modes to achieve optical switching using the changeable radius cavity within the middle of the PhC-mesh [26,27]. An optical switch using two linear waveguides and a circular geometry of the PhCs is studied in [28], using temperature as the variable quantity for the switching mechanism in terms of a shift in the resonance wavelength. The use of broken mirror symmetry to obtain asymmetric Fano-shapes is described in [29], utilizing partially transmitting material within the waveguide to propose an optical switching device and its prospects [30]. A four-port optical switch is investigated in [31], using the square lattice of the PhCs and rods of gallium phosphide with a medium composed of air; metallic embedding nanowires [32], the use of the Finite Difference Time Domain (FDTD) approach, and a thermo-optic effect with a super-ellipse shape [33] have also been studied. A similar idea is perceived in [34], using the plane-wave expansion (PWE) approach to estimate the bandgap of an optical switching device with a discussion of its applications in optical comparators [35]. Based on the design of the 2D PhC-cavity structure and its transmission spectra, an optical switching device is investigated by using the Kerr effects to produce a shift in its resonant wavelength given in [36]; the use of III-V nanowires on a silicon (Si) platform for the purpose [37], strong carrier-induced nonlinearity [38], defect rods made of doped glass [39], linear PhCs [40], and a non-linear cavity coupled to input and output of the waveguide [41] have also been studied. 

Similarly, using quantum dots (QDs) to observe the mechanisms of optical switching and achieve higher rates of transmittance and flat bandwidth using latch function is premediated in [42], and are utilized through a Symmetric Mach Zehnder (SMZ) for the purpose in [43]. Plasmonics effects are exploited for optical switching by applying a strip of a graphene layer onto a structure of PhCs in [44], and the mechanism is investigated using graphene rods in [45]. An approach of using concepts of mott phase change material in PhC-based structures responsible for the shifting of the optical bandgap is investigated in [46], and polymer waveguides with high thermos-optic and electro-optic properties are used in in [47]. Similarly, a 3D Si-opal composite is reported in [48], with a pump-probe technique in [49], and 1D graphene-based plasmonics crystals are used in [50] to efficiently determine the effects of optical switching. In addition, using two PhC cavities within one arrangement is reported in [51], using a waveguide between them. Thus, coupling a probe signal in the waveguide affects the field distribution and quality factor of both of the PhC-cavities, enabling the structure to be comprehended as an optical switch. Soft PhCs in the form of chiral liquid crystals are also used to obtain certain properties of the optical switch, as described in [52], while are obtained through effects of physics in terms of parity–time symmetry and topological insulators in [53], and by using phase change nanomaterials [54], air-trench [55] and carrier diffusion and recombination processes in PhCs [56], with the design based on 3D Micro-Electro-Mechanical-Systems (MEMS) reported in [57].

**Figure 1:**
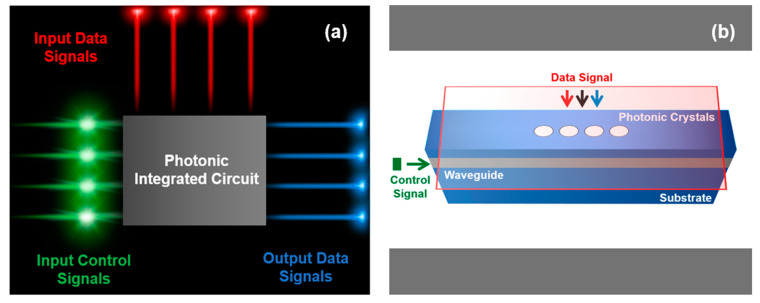
(**a**) Photonic integrated circuit. (**b**) conceptual diagram of the 3D-optical switch.

This research investigates the design of a 3D-optical switch working in the near-infrared region (NIR), suitable for communication systems. Moreover, the design is implemented using the 3D-FDTD domain and the interference phenomenon between the GMR modes and index-guided modes inside a slab-waveguide PhC structure. For this purpose, the proposed device uses two optical signals, i.e., data and control. The data signal is integrated into the optical structure utilizing the out-of-plane method, i.e., GMR, and is amplified or de-amplified using a control signal, which is index-guided into the slab-waveguide structure. Figure 1a imitates the theoretic 3D structure of the optical switch, presenting an innovative, compact, easy-to-fabricate, and implementable device. Likewise, due to the lower absorption of dielectric materials, they are suitable for broad spectral ranges; in contrast, semiconductors have higher material costs and absorption levels, making their use and fabrication difficult in densely populated optical integrated circuits, as investigated in previous studies. Figure 1b illustrates the use of the proposed optical switch in an optical integrated circuit. Therefore, it can find applications in the field of optical integrated components, filters, sensors, quantum computing, and communication systems. 

## 2. Simulation Approach

The 3D-FDTD simulation of the proposed structure is performed using the MIT Electromagnetic Equation Propagation (MEEP) software [58], an open-source platform, based on the FDTD domain [59]. Therefore, during execution, the desired arrangement is initially transformed into a finite-arrangement of grids, known as YEE grids [60], to compute the Electric ‘E’ field and Magnetic ‘B’ field based on Maxwell’s equations. Moreover, FDTD is advantageous in terms of computing the response of the system in a single run (using a short Gaussian pulse), providing the user with the propagation of the wave both in the near-field and far-field, especially inside complex structures such as PhCs [61]. Apart from these advantages, it requires fewer computational resources, less memory, and less time as compared to frequency-domain-based simulations [62]. 

## 3. Designing Parameters

The optimized designing parameters of the proposed 3D-optical switch are given in Table 1 in terms of lattice-constant ‘a’ chosen as (a = 1 µm), serving two purposes, i.e., to enable the design of the device scalable to any range of wavelength and enable its operation in the telecommunication window around 1.55 µm, using cylindrical-shaped-based PhC elements filled with air having a refractive index of n_air_ = 1. 

**Figure 2:**
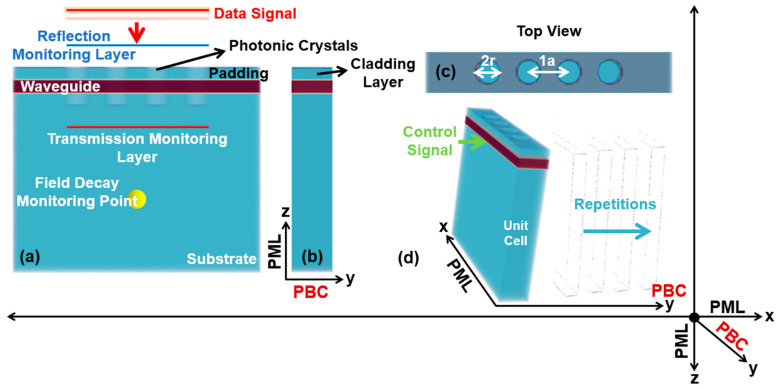
Infinite structures of the 3D-optical switch. (**a**) The view of the 3D-optical switch along *x*-axis showing the data signal, reflection, and transmission monitoring layers, and field decay monitor point. (**b**) Cross-section view along *y*-axis. (**c**) Top view along *x*-axis. (**d**) A unit cell of the device showing the direction of the control signal.

Figure 2 imitates the design of the proposed 3D-optical switch using niobium pentaoxide (Nb_2_O_5_) as the waveguiding material, with a refractive index of n_wav_ = 2.2 [25], which is deposited on a substrate made up of silicon dioxide (SiO_2_) with a refractive index of n_sub_ = 1.5 [27], along a cladding layer of the SiO_2_ placed on the topmost side of the waveguiding layer. Moreover, it represents the strip model of the 3D-optical switch, based on which, the investigations are performed and two finite models are computed in the end. 

For the data signal, a Gaussian time profile source is used, located at a range of 2.7a above the waveguide, with a similar profile source for the control signal index guided in the optical structure from the lateral side using a user definite sum of frequencies ‘n_f_’ as (n_f_ = 550) for both of the sources. Similarly, the transmission and reflection monitoring layers are positioned at a range of 1.0a below and above the waveguide, respectively, to compute the electromagnetic (EM) fields. For the purpose of terminating the simulation process, a field decay monitoring point is positioned at a user-definite distance of 1.5a. To observe the behavior and prevent back-and-forth of the EM fields at the boundaries of the structure, the Perfectly Matched Layers (PML) are implemented along the x-z axes. Moreover, to save time and computational resources, the periodic boundary condition (PBC) is used on the *y*-axis. However, the condition of the PBC is removed and is replaced by PML during the simulation of the finite structures of the 3D-optical switch. 

## 4. Results

**Figure 3:**
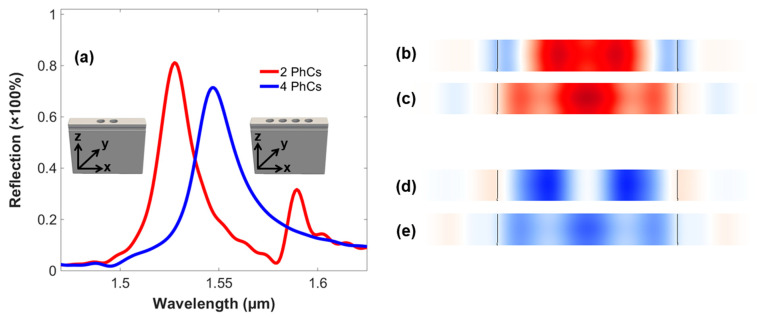
(**a**) The output reflection spectra of the infinite models of the 3D-optical switch comprising 2 and 4 PhC elements (along the *x*-axis). (**b**) Electric field confinement by the 2-PhC-element-based structure. (**c**) Electric field confinement by the 4-PhC-element-based structure. (**d**) Magnetic field confinement by the 2-PhC-element-based structure. (**e**) Magnetic field confinement by the 4-PhC-element-based structure.

The 3D-FDTD simulation of the optical switch is performed in this research using the stripe model criterion. For this purpose, the first step is determining the number of PhC elements using a single source, i.e., the data signal only, to tune the GMR modes around the resonant wavelength, i.e., 1.55 µm. For this purpose, Figure 3a imitates the output reflection spectra of the structure comprising two and four PhC elements. Therefore, it can be seen that the structure based on two PhC elements has a higher intensity of reflection peaks, as compared to the four-PhC-element-based structure. However, it is worth noting from the output spectra that the resonant wavelength of the two-PhC-element-based arrangement is around 1.528 µm, while is at 1.547 µm in the four-PhC-element-based structure. Similarly, Figure 3b,c and Figure 3d,e show the Electric ‘E’ and Magnetic ‘B’ fields’ confinement in the structures comprising two and four PhC elements, respectively. 

Therefore, comparing the arrangements based on the number of PhCs, the two-PhC-based structure has the advantage of coupling the GMR modes more efficiently, which is due to the lesser change in the effective refractive index of the waveguide. However, the modes are not confined to the required resonant wavelength of the device. In contrast, the four-PhC-based arrangement having a higher number of PhCs elements produces lower values of reflection peaks due to changes in the refractive index of the waveguide and an increased fill factor (air) inside the optical structure, resulting in lower intensities. However, the increase in the number of PhC elements does produce confined GMR modes near the resonant wavelength of the device. This can be attributed to the increased phenomenon of interference between the incident modes and the leaky modes inside the optical device. Table 2 presents detailed comparisons between the two- and four-PhC-based structures in terms of resonant wavelength, the percentage of reflection peak, linewidth, and quality factor, i.e., calculated by using Equation (1):

(1) Quality factor=reflection peak/linewidth

Apart from the change in reflection peaks and resonant wavelength, there is a visible difference in the linewidth and the quality factor between these two structures. Corresponding to the linewidth, it remains low in the two-PhC-element-based arrangement and is a bit on the higher side in the four-PhC-element-based structure. Similarly, the quality factor is higher in the former structure, and vice versa. However, each arrangement possesses its pros and cons, which can be entangled during the design of the 3D-optical switch. As an acknowledgeable fact, the number of PhC elements can be increased, as it may create more confined GMR modes using data signal only, but it is limited to four PhC elements only, due to the fact that it will in turn reduce the outcomes of the control signal on the output of the data signal. In other words, it will reduce the switching properties of the device equivalent to the depletion region of the electronic transistor. Apart from these, further structuring in the 3D domain relating to an increase in the number of PhC elements is also limited by the computational resources in terms of speed and memory. As a result, achieving the unity factor in the output spectra is hindered. Similarly, the propagation losses incurred by the control signal due to gratings also contribute to this and are added to the absorption losses of the materials. Henceforth, both of the arrangements are investigated in relation to the operations of optical switching, resulting in two 3D finite structures of the device i.e., based on two and four PhC elements in the end. 

### 4.1. Study of Optical Switching Phenomenon by Changing the Number of PhC Elements Using Data and Control Signals Simultaneously

To investigate the phenomena of the optical switching in 3D PhC-element-based structures, i.e., two and four PhC elements, the control signal is executed along with the data signal. Moreover, the wavelength of the control signal is also varied to investigate its detailed response on the output of the data signal. Table 3 shows the criterion used in changing the wavelength of the control signal with regard to the data signal. 

**Figure 4:**
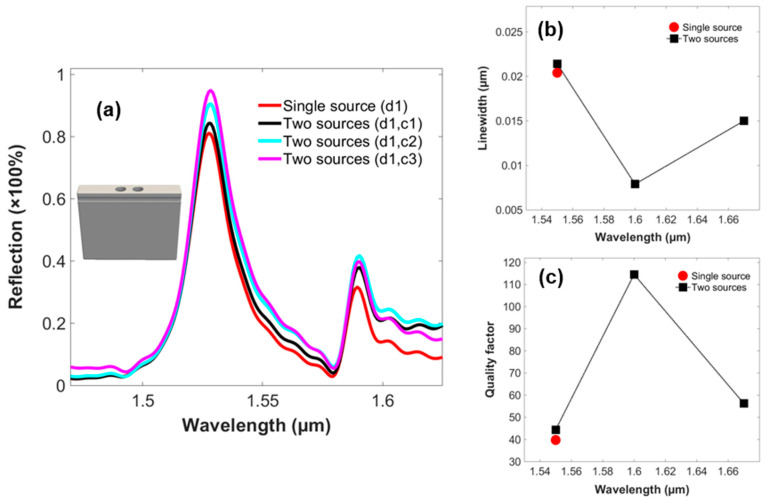
(**a**) Output reflection spectra of the data signal relating to infinite model of the 2-PhC-element-based structure, investigating the phenomenon of optical switching relating to varying wavelength of the control signal. (**b**) Linewidth. (**c**) Quality factor.

The effects of the control signal are given in the reflection spectra of the two-PhC-element-based structure in Figure 4a. It investigates the increase in the percentage of the reflection peaks as the wavelength of the control source is varied from 1.67 µm to 1.55 µm and vice versa. Therefore, it studies a prominent change in the control source at a wavelength of 1.55 µm. Figure 4b illustrates the alteration in the linewidth of the structure as the wavelength of the control signal is varied, reporting the lowest value of 0.0079 µm at a wavelength of 1.60 µm. Figure 4c probes the quality factor of the structure, achieving a maximum value of 112 at a wavelength of 1.60 µm of the control signal. Thus, the GMR modes are confined and offer higher selectivity for the two-PhC-element-based structure.

**Figure 5:**
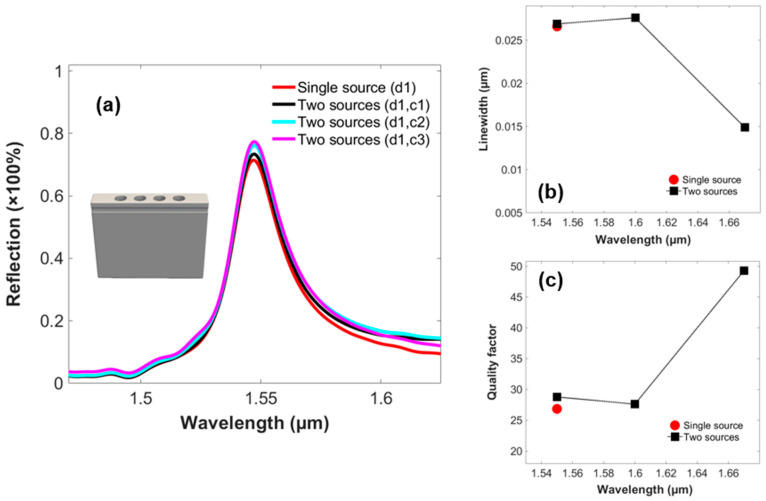
(**a**) Output reflection spectra of the data signal relating to infinite model of the 4-PhC-element-based structure, investigating the phenomenon of optical switching with regard to varying wavelength of the control signal. (**b**) Linewidth. (**c**) Quality factor.

Similarly, Figure 5a investigates the four-PhC-element-based structure for optical switching action. Therefore, it can be seen that implementing the control signal along the data signal increases the coupling of the GMR modes and energy further into the optical structure, and as a result, reaches nearer to the desired resonant wavelength of the structure, i.e., 1.55 µm. Moreover, it achieves higher percentages of reflection spectra when the wavelength of the control signal is varied from 1.67 µm to 1.55 µm and is maximum when this wavelength is equivalent to the wavelength of the data signal, i.e., 1.55 µm. Figure 5b imitates the variation in the linewidth of the structure based on four PhC elements, achieving a minimum value of around 0.0149 µm at a wavelength of 1.67 µm of the control signal. Figure 5c reports the variation in the quality factor with regard to change in the wavelength of the control signal. Therefore, it can be concluded that the structure achieves the maximum value of quality factor, i.e., 49.28, at a 1.67 µm wavelength of the control signal. Therefore, the optimum figure of the wavelength of the control signal is 1.60 µm and 1.67 µm for the two- and four-PhC-element-based structures, respectively. Table 4 shows the detailed responses of both of the topologies in terms of resonant wavelength, reflection, linewidth, and quality factor. 

Correspondingly, the phenomenon which is responsible for the change in the output of the data signal is due to non-linear optical effects. Elaborating upon the non-linear effects, when a data signal and the control signal are coupled into the optical structure comprising the PhC elements, the frequency of the output signal is doubled, which in turn helps in the amplification of the data signal of the optical device and occurs when there are higher-order modes in the E field. Apart from these, the effects depend on other factors as well, i.e., the propagation of the wave in the medium, second harmonic generations, and conservation laws for photons. Therefore, the non-linearity factor of the device leads to the generation of the new frequency components and, as a result, leads to the amplification of the data signal. Moreover, it is worth noting here that as the frequency of the control signal is increased, it results in the amplification of the data signal in an ascending order. However, the applied frequency of the control signal, i.e., 1.60 µm, produces a narrower linewidth and highest quality factor in both of the cases of the PhC-based structures. This can be attributed to the resonance factor of all the frequencies in the structure inducing the GMR modes and coupling of energy further into the optical structure. Considering the highest percentage of amplification achieved, with an applied frequency of the control signal of 1.55 µm, the linewidth is wider and the quality factor is lower for this case. Although the frequency of both sources, i.e., data and control, are the same, due to the non-linear nature of the device, this leads to a degrading linewidth and quality factor of the device. Henceforth, by applying the control signal, the linewidth of the device is narrowed and the quality factor is increased, helping in the enhancement of the GMR modes, the coupling of energy, and the selectivity of the device. 

### 4.2. Finite Structures of the 3D-Optical Switch Based on Two and Four PhC Elements 

**Figure 6:**
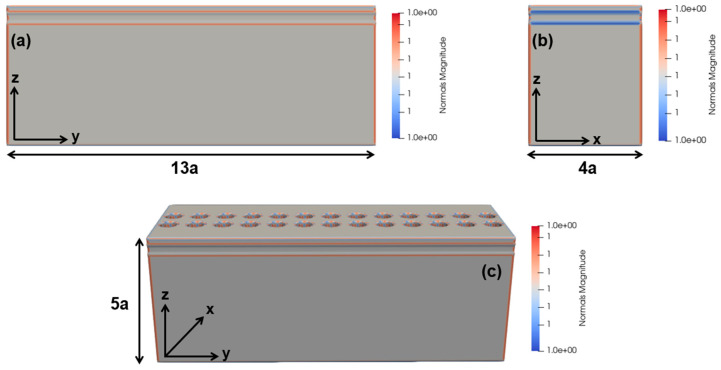

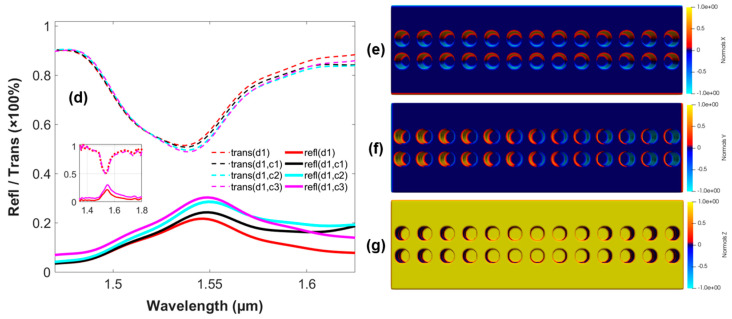
Finite model of the 3D-optical switch comprising the 2 PhC elements along *x*-axis. (**a**) Lateral view of the optical switch along *y*-axis. (**b**) Cross-sectional view along *x*-axis. (**c**) Finite model representation along x, y, and z directions. (**d**) Output reflection and transmission spectra of the finite model signifying the properties of the optical switching. (**e**) Field confinement (*x*-axis). (**f**) Field confinement (*y*-axis). (**g**) Intensity of data signal on the surface of the optical switch (*z*-axis).

The next step is to investigate and draw finite structures of the 3D-optical switch by removing the PBC, previously implemented on the *y*-axis, in the case of the striped model. Moreover, instead, the PML is executed on all of the three axes for the finite realization of the device. For this purpose, the two-PhC-element-based structure is realized first, as shown in Figure 6a–c, depicting the length, width, and height of the device. 

Therefore, for the optical switching mechanism, the structure is simulated using data and control signals simultaneously, with reflection and transmission spectra imitated in Figure 6d. So, it can be shown from the spectra that by implementing the control signal and varying its wavelength from 1.67 µm to 1.55 µm, the output of the data signal tends to increase and vice versa, thus, helping in the confinement of GMR modes and energy. However, it is worth noting here that the intensity and coupling of energy of the resonant modes is lower to a certain degree in comparison to the infinite striped model of the two-PhC-element-based structure. Henceforth, it can be increased and the desired level can be achieved by increasing the number of PhC elements along the *y*-axis, which is limited by the computational resources at present. Figure 6e,f, show the field confinement of the structure along the x and y directions, and Figure 6g depicts the intensity and confinement of the data signal only on the surface of the structure. 

**Figure 7:**
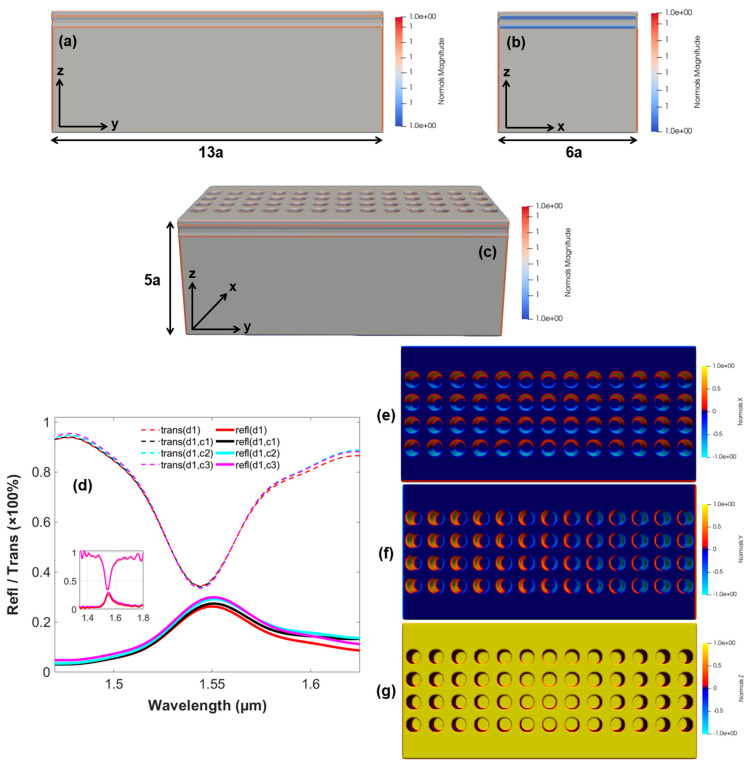
Finite model of the 3D-optical switch comprising the 4 PhC elements along *x*-axis. (**a**) Lateral view of the optical switch along *y*-axis. (**b**) Cross-sectional view along *x*-axis. (**c**) Finite model representation along x, y, and z directions. (**d**) Output reflection and transmission spectra of the finite model signifying the properties of optical switching. (**e**) Field confinement (*x*-axis). (**f**) Field confinement (*y*-axis). (**g**) Intensity of data signal on the surface of the optical switch (*z*-axis).

Similarly, the structure of the 3D-optical switch based on the arrangement of the four PhC elements is imitated in Figure 7a–c, reporting the length, width, and height of the finite model, respectively. Moreover, the optical switching action is shown in Figure 7d using the reflection and transmission spectra of the structure, executing the control signal with a varying wavelength in the range, i.e., 1.67–1.55 µm along the data signal simultaneously. Therefore, it can be seen that increasing the units of PhC elements increases the confinement of the GMR modes and coupling of energy further into the optical device. However, the increase in the number of the PhC elements (*x*-axis) has been reported to have adverse effects on the optical switching properties of the device. Therefore, the number of PhC elements is limited to four only, according to this research. Moreover, to increase the coupling and confinement of the GMR modes, the number of PhC elements can be increased along the *y*-axis, which will result in the reduction in the compactness of the device as a whole. Similarly, the field confinement by the structure along the x and y directions is shown in Figure 7e,f, with Figure 7g depicting the intensity of the data signal only on the surface of the optical structure.

The designed optical switching device can be used in a number of applications relating to the control and utilization of light. One specific application of the device is in the field of biotechnology, i.e., testing the numerous components in the blood. This can be achieved as the device will produce spectra of different intensities and values, beneficial in determining its composition. In this case, if the intensity of a specific component is lower than the threshold value, it can be enhanced by the execution of the control signal for this purpose. Similarly, the same phenomena of testing can be utilized to investigate the different components of water as well, as safe for drinking and as recommended by the World Health Organization (WHO). Moreover, considering communication systems, the designed device can be used as an amplification device, acting as a repeater and helping in the overall transmission of optical data over long-haul communications.

## 5. Discussion

The modeling of a 3D-optical switch is still an innovative idea, having qualities such as compactness, ease of fabrication, and being implementable in PICs. Therefore, an optical switching idea presented in [25] uses a control signal for the amplification of the data signal in numeral structures composed of a variable number of PhC elements. Moreover, another structure of the optical switch, as presented in [25,26], uses 11 PhC elements along with a variable PhC cavity realized at the start of the PhC mesh to modulate the GMR modes. Apart from these, a variable PhC cavity along the outcomes of the control signal on the output of the data signal and tunning of the GMR modes are modeled in [27]. To achieve efficient optical switching, different PhC-element-based structures are used. However, one common path is followed in all of these investigations, i.e., using the 2D simulation domain. Apart from these, optical switching is reported in [63], using the plasmonics characteristics of graphene having a Si-core-based waveguide. The refractive index of the graphene clad is varied with a low-intensity pulse, in contrast, which modifies the characteristics of the device, and as an outcome, the aspects of the optical switch are achieved. Table 5 reports the details of the previous studies in comparison with the current research. 

## 6. Proposed Fabrication Process

**Figure 8:**
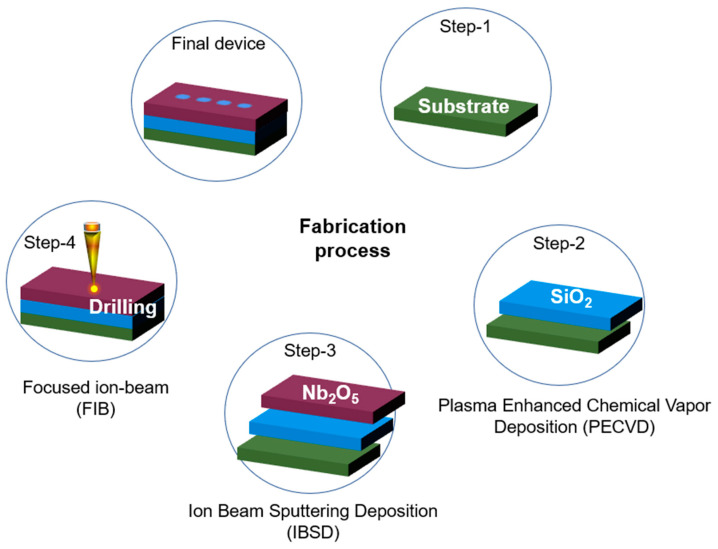
The stepwise fabrication process of the reported optical switching device.

The proposed optical switching device can be realized using the conventional fabrication techniques used for optical integrated components, as shown in Figure 8. In the first step, Plasma-Enhanced Chemical Vapor Deposition (PECVD) can be used for the deposition of SiO_2_ to form the base of the structure [64]. In the second step, the waveguiding layer, i.e., Nb_2_O_5_, can be deposited over the layer of SiO_2_ using the method of Ion Beam Sputtering Deposition (IBSD) [65]. In the last step, an array of PhC elements can be structured using the Focused Ion-Beam (FIB) platform [66]. 

## 7. Conclusions

In this study, a 3D-FDTD depiction of an optical switch is investigated. The switching action is studied by utilizing two sources, namely the data signal and the control signal in a dielectric slab-waveguide structure having an array of PhC elements. The data signal is coupled into the device using GMR, which filters the data signal at the same time, while the control signal is index-guided into the optical structure, which is responsible for the optical switching and amplification of the data signal. The switching and amplification operation is fine-tuned by varying the central wavelength of the control and structural parameters of the device. The structural specifications of the device are optimized in terms of the radius and number of periods of the PhCs, i.e., two PhC elements and four PhC elements (along the *x*-axis). Likewise, two topologies are used, i.e., structuring of the device using an infinite modeling mechanism, where a specific chunk of the device (along the *y*-axis in this research) is repeated using the condition of PBC, to save time and computational resources. Secondly, a finite modeling mechanism is used, where a finite structure is concluded based on the results of the infinite structuring mechanism. Therefore, in the case of the arrangement composed of two PhC elements, it produces pronounced effects of optical switching with high peaks in reflection spectra, narrow linewidth, and high-quality factor. However, it is not very effective in achieving the required resonant wavelength of the device. Similarly, implementing the four-PhC-element-based structure achieved the lower coupling of energy having a wider linewidth and decreased quality factor as compared to the two-PhC-element-based structure. However, the device can efficiently achieve optical switching at the desired wavelength range of the device, i.e., 1.55 µm. As a result, an amplification of about 13.75% with a linewidth of 0.0079 µm and a quality factor of 114.58 is attained. Considering the cases of finite modeling, i.e., two PhC elements and four PhC elements (along the *x*-axis), the expected results of the infinite structuring mechanism have been reported. The design can be improved further by increasing the units of the PhC elements (along the *y*-axis), which is currently limited by the computational resources. Henceforth, the proposed design can be efficiently used in the fields of PICs, communications systems, biomedical sensors, and programmable photonics. 

## Figures and Tables

**Table 1:** Augmented parameters of the 3D-optical switch.

Parameters	Symbol	Value
Lattice constant	a	1 µm
Radius of PhC elements	r	0.300a
Thickness of waveguide	w_thick_	0.330a
Thickness of cladding	-	0.300a
Thickness of Perfectly Matched Layer	PML	3.0a, (along *x* and *z*) axes
Refractive index of the substrate	n_sub_	1.5
Refractive index of the waveguide	n_wav_	2.2
Field decay monitoring point	-	1 × 10^−3^
Padding	P_add_	2.0a
Periodic boundary condition	PBC	Along *y*-axis
Resolution	-	20
Smoothing factor	-	0.05
Grid size	-	0.52

**Table 2:** Properties achieved by the infinite models of the 3D-optical switch comprising of 2 and 4 PhC elements.

Number of PhC Elements	Resonant Wavelength (µm)	Reflection Peak (%)	Linewidth (µm)	Quality Factor -
2 PhC elements	1.528	81.13	0.0204	39.76
4 PhC elements	1.547	71.43	0.0266	26.85

**Table 3:** Names and differences in wavelengths of the data and control signal.

Type of Source	Name of Source			
	d1	c1	c2	c3
Data signal	1.55 µm	-	-	-
Control signal	-	1.67 µm	1.60 µm	1.55 µm

**Table 4:** A detailed comparison between the infinite models of the 3D-optical switch based on 2 and 4 PhC elements.

**2 PhC Elements**				
**Sources**	**d1**	**c1**	**c2**	**c3**
Resonant wavelength (µm)	1.528	1.528	1.528	1.528
Reflection (×100%)	0.8113	0.8434	0.9052	0.9488
Linewidth (µm)	0.0204	0.0150	0.0079	0.0214
Quality Factor	39.77	56.23	114.58	44.34
**4 PhC Elements**				
**Sources**	**d1**	**c1**	**c2**	**c3**
Resonant wavelength (µm)	1.547	1.547	1.547	1.547
Reflection (×100%)	0.7143	0.7343	0.7623	0.7741
Linewidth (µm)	0.0266	0.0149	0.0276	0.0269
Quality Factor	26.85	49.28	27.62	28.78

**Table 5:** Comparison of the previous research studies with the current investigation for the design of the 3D-optical switch.

Design	Resonant Wavelength (µm)	Reflection (×100%)	Simulation Domain	Linewidth (µm)	Quality Factor	Research Work
SiO_2_ + Nb_2_O_5_ + PhCs (air)	1.544	0.9592	2D	0.0696	11.87	[25]
SiO_2_ + Nb_2_O_5_ + PhCs (air)	1.534	0.8695	2D	0.0727	-	[26]
SiO_2_ + Nb_2_O_5_ + PhCs (air)	1.542	0.9518	2D	0.0652	12.96	[27]
SiO_2_ + Nb_2_O_5_ + PhCs (air)	1.528	0.9488	3D	0.0079	114.58	This work

## Data Availability

Not applicable.
